# COX-2 upregulation by tumour cells post-chemotherapy fuels the immune evasive dark side of cancer inflammation

**DOI:** 10.15698/cst2022.09.271

**Published:** 2022-08-16

**Authors:** Charlotte R. Bell, Santiago Zelenay

**Affiliations:** 1 Cancer Inflammation and Immunity Group, Cancer Research UK Manchester Institute, The University of Manchester, Alderley Park, Macclesfield, UK.; 2 The Lydia Becker Institute of Immunology and Inflammation, The University of Manchester, Manchester, UK.

**Keywords:** chemotherapy, immune checkpoint blockade therapy, cancer inflammation, COX-2, prostaglandin E_2_

## Abstract

Cytotoxic therapies, such as chemotherapy and radiotherapy, are mainstays of cancer treatment for both early and unresectable, advanced disease. In addition to debulking the tumour mass through direct killing of proliferating tumour cells, these treatments can promote tumour control via immune-stimulating effects. Nonetheless, chemoresistance and tumour relapse remain huge clinical problems, suggesting that induction of anti-cancer immunity post-cytotoxic therapy is often weak, not durable and/or overcome by immune evasive mechanisms. In our recent study (Nat Commun 13:2063), we demonstrate that cancer cell-intrinsic activation of the cyclooxygenase (COX)-2/prostaglandin E_2_ (PGE_2_) pathway post-chemotherapy treatment is a prevalent phenomenon which profoundly alters the inflammatory properties of the treated cancer cells. Of particular translational relevance, our findings support a model whereby upregulation of COX-2 expression and activity post-chemotherapy impairs the efficacy of the combination of PD-1 blockade and chemotherapy. Accordingly, pharmacological inhibition of COX-2 with celecoxib, an anti-inflammatory drug already used clinically, unleashed tumour control in preclinical models when given alongside chemoimmunotherapy combinations.

Immune checkpoint blockade (ICB) therapies have transformed cancer treatment but only a fraction of patients experience benefit, especially long-term. Combinations with other treatment modalities are therefore actively being evaluated to improve patient outcomes. In particular, the combination of ICB with standard of care chemotherapy regimens has shown promise for multiple indications, leading to approvals for first-line treatment in different cancer types including non-small cell lung cancer, triple negative breast cancer (TNBC) and bladder cancer. However, clinical trial results have been mixed, with chemoimmunotherapy combinations in some cases failing to provide a benefit above standard of care chemotherapy. The design of more effective rational combinations requires a better understanding of the underlying basis for intrinsic and acquired therapy resistance.

Cancer-associated inflammation can play a dual role in tumour progression, with the potential to foster or restrain tumour growth. Therapeutic strategies that modulate the inflammatory tumour microenvironment (TME) to facilitate immune-mediated tumour control could therefore be valuable partners to chemoimmunotherapy combinations. Our previous work has underscored the cyclooxygenase (COX)-2/prostaglandin E_2_ (PGE_2_) axis as a nodal protumourigenic inflammatory pathway often co-opted by cancer cells to drive immune evasion and resistance to ICB. In this latest study, we set out to investigate its contribution to inflammation and tumour immunity during chemotherapy treatment. Of interest, previous studies had already implicated PGE_2_ release from dying cancer cells post-cytotoxic therapy in stimulating cancer cell proliferation and tumour repopulation. We sought to establish the prevalence, underlying mechanisms, and consequence of PGE_2_ upregulation for the inflammatory properties of cancer cells post-cytotoxic therapy.

We first characterised activation of the COX-2/PGE_2_ pathway in chemotherapy-treated cancer cells and showed that transcriptional upregulation of the COX-2 gene and downstream PGE_2_ release occurs across multiple murine cancer cell lines, but notably only in cancer cells with basal COX-2 expression. By analysing a publicly available dataset of 60 human cancer cell lines treated with different chemotherapy drugs, we confirmed this phenomenon is conserved in mice and man regardless of the tumour tissue of origin. Chemotherapy-driven PGE_2_ release strictly relied on the enzymatic activity of COX-2, as it was fully prevented by addition of the selective COX-2 inhibitor celecoxib. Furthermore, transcriptional upregulation of *Ptgs2* (encoding COX-2) post-chemotherapy was essential for enhanced PGE_2_ production. Thus, cancer cells genetically engineered to express COX-2 from an unrelated constitutive promoter were unable to upregulate the pathway in response to chemotherapy. These findings have important clinical implications, as measuring baseline activity of the COX-2/PGE_2_ pathway in tumours pan-cancer type could help identify patients most likely to benefit from the addition of a COX-2 inhibitor to chemoimmunotherapy regimens.

We generated a reporter cell line to study the kinetics of COX-2 transcriptional upregulation alongside treatment effects on cancer cell growth by live cell imaging. The reporter cells express destabilised GFP, a short half-life version of GFP, under the control of the endogenous *Ptgs2* promoter. After confirming the increase in GFP fluorescence by these reporter cells post-chemotherapy closely reflected upregulation of COX-2 mRNA transcription, we utilised them to screen a library of 1280 market-approved drugs. Remarkably, all classes of chemotherapeutic agents led to increased COX-2 transcription when used at concentrations that induced cancer cell growth arrest, indicating that activation of the COX-2/PGE_2_ pathway happens independently of the drug mechanism of action. Noteworthy, both chemotherapy drugs reported to stimulate, and not stimulate, immunogenic cell death (ICD) similarly increased COX-2 expression and PGE_2_ synthesis, excluding this phenomenon as major contributor to the differential ability of ICD and non-ICD inducers to stimulate cancer immunity.

Interrogating the mechanism underlying COX-2/PGE_2_ upregulation post-chemotherapy through inhibitor and siRNA-based approaches, we excluded a major role for caspase activity, reactive oxygen species or various transcription factors known to bind to the *Ptgs2* promoter region, including NF-κB, C/EBPβ, Sp1 and AP-1. Furthermore, we demonstrated that this response occurs prior to activation of the apoptosis executioners caspase-3/-7 but coincides with the halt in cancer cell proliferation. Indeed, no transcriptional COX-2 upregulation was observed for any of the diverse compounds tested in the library screen unless they also concomitantly promoted cancer cell arrest. Accordingly, analysis of an available transcriptome dataset of multiple human cancer cell lines treated with different chemotherapy agents indicated that the increase in COX-2 transcription correlates with the level of growth inhibition induced by the drug. Given how prevalent the increase in COX-2 expression post-chemotherapy was across mouse and human cancer cells and the variety of treatments that could trigger it, we speculate that there is a widely conserved, yet unidentified, cell stress response pathway that underlies the acute upregulation of *Ptgs2* transcription. The full characterisation of this pathway and whether it also contributes to COX-2 upregulation post-cytotoxic treatment in non-tumour cells warrants further investigation.

To determine the impact of increased COX-2/PGE_2_ pathway activity on the inflammatory properties of cancer cells post-chemotherapy *in vivo,* we assessed the recruitment of immune cells and production of soluble inflammatory mediators after injecting chemotherapy-treated cells into the peritoneum of mice. This experimental system, previously used to study the inflammatory response to dying cells, allowed us to delineate the response driven by the treated cancer cells, without the confounding effects that systemic chemotherapy would have on non-tumour cells. Using this approach, we showed that cancer cells which upregulate COX-2 expression post-chemotherapy stimulate markedly enhanced recruitment of neutrophils and monocytes or production of soluble factors compared with control untreated cancer cells. Notably, cancer cells which lacked or expressed basal levels of COX-2 but could not upregulate its transcription post-chemotherapy behaved similar to untreated cells, uncovering the increase in COX-2 as a major determinant of the inflammatory properties of chemotherapy-treated cancer cells.

Given these findings and our previous work highlighting a crucial contribution for cancer cell-intrinsic COX-2/PGE_2_ activity to immune escape, we next evaluated whether pharmacological inhibition of COX-2 would alter the efficacy of chemoimmunotherapy combinations. Clinical trials evaluating the addition of COX-2 inhibitors to cytotoxic therapy regimens have historically produced mixed results. Since inhibition of the COX-2/PGE_2_ pathway can efficiently enhance responses to ICB in multiple pre-clinical tumour models, it may be that additional stimulation of the immune system is required to observe a therapeutic benefit from chemotherapy and COX-2 inhibitor combinations in certain contexts. Our findings are in line with this speculation, as mice bearing poorly immunogenic tumours formed by 4T1 breast cancer cells failed to respond unless treated with the triple drug combination of chemotherapy, PD-1 blockade and COX-2 inhibition. The triple combination reduced the accumulation of neutrophils and monocytes, often associated with immunosuppression and ICB resistance, and increased the proportion of tumour-infiltrating IFNγ-producing T cells, indicating mobilisation of an enhanced anti-cancer immune response. Likewise, in a colorectal cancer model partially sensitive to chemoimmunotherapy, COX-2 inhibition further improved tumour control, indicating that pharmacologically targeting the COX-2/PGE_2_ pathway could benefit tumours both sensitive or refractory to chemoimmunotherapy.

Metastatic spread remains a major clinical challenge and multiple trials are examining the benefit of chemoimmunotherapy combinations for the prevention and treatment of cancer metastasis. We utilised the metastatic orthotopic 4T1 model of TNBC to explore whether the addition of a COX-2 inhibitor could improve the efficacy of chemoimmunotherapy in an adjuvant therapy preclinical model. Mammary tumours were surgically removed prior to the initiation of therapy, and the rate of local tumour reoccurrence and lung metastasis monitored. In this experimental setting, the addition of a COX-2 inhibitor alongside chemoimmunotherapy was again essential to limit tumour relapse and spontaneous metastasis after surgery.

In conclusion, our findings support a model whereby increased COX-2 expression and the resulting augmented PGE_2_ synthesis, by modulating the inflammatory features of cancer cells post-cytotoxic treatment, limit T cell-mediated tumour immunity (**Fig. 1**). Therefore, pharmacologically targeting the COX-2/PGE_2_ axis could represent a viable strategy to unleash the efficacy of combinations of cytotoxic therapy and immunotherapy.

**Figure 1:**
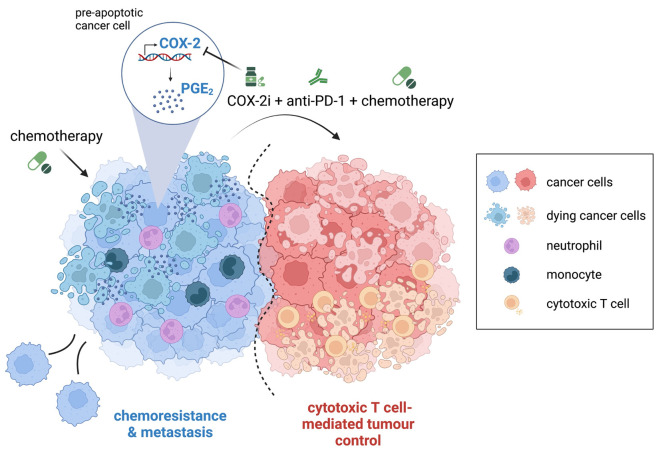
FIGURE 1. Cytotoxic therapy modulates tumour-associated inflammation via activation of the COX-2/PGE_2_ pathway. Chemotherapy upregulates COX-2 transcription and downstream PGE_2_ production in dying cancer cells, inducing an inflammatory response that limits the efficacy of chemoimmunotherapy combinations. Inhibition of COX-2 alongside chemoimmunotherapy treatment enhances immune-mediated tumour growth control and prevents metastasis in pre-clinical models.

